# Afferent neurons of the kidney with impaired firing pattern in inflammation – role of sodium currents?

**DOI:** 10.1007/s00424-023-02852-6

**Published:** 2023-09-06

**Authors:** Nena Lale, Tilmann Ditting, Karl F. Hilgers, Peter Linz, Christian Ott, Roland E. Schmieder, Mario Schiffer, Kerstin Amann, Roland Veelken, Kristina Rodionova

**Affiliations:** 1 Department of Internal Medicine 4 Nephrology and Hypertension, Friedrich-Alexander University Erlangen, 91054 Erlangen, Germany; 2 Department of Internal Medicine 4 – Nephrology and Hypertension, Paracelsus Private Medical School Nuremberg, Nuremberg, Germany; 3 Department of Radiology, Friedrich-Alexander University Erlangen, 91054 Erlangen, Germany; 4 Department of Nephropathology, Friedrich-Alexander University Erlangen, 91054 Erlangen, Germany

**Keywords:** Renal afferent nerve, Sodium currents, Voltage-gated sodium channels, Tonic, Phasic, Firing pattern, Chemokine

## Abstract

Peripheral neurons with renal afferents exhibit a predominantly tonic firing pattern of higher frequency that is reduced to low frequencies (phasic firing pattern) in renal inflammation. We wanted to test the hypothesis that the reduction in firing activity during inflammation is due to high-activity tonic neurons switching from higher to low frequencies depending on altered sodium currents. We identified and cultivated afferent sensory neurons with renal projections from the dorsal root ganglia (Th11-L2). Cultivated neurons were incubated with the chemokine CXCL1 (1,5 nmol/ml) for 12 h. We characterized neurons as “tonic,” i.e., sustained action potential (AP) firing, or “phasic,” i.e., < 5 APs upon stimulation in the current clamp. Their membrane currents were investigated in a voltage clamp. Data analyzed: renal vs. non-renal and tonic vs. phasic neurons. Renal afferent neurons exposed to CXCL1 showed a decrease in tonic firing pattern (CXCL1: 35,6% vs. control: 57%, *P* < 0.05). Na^+^ and K^+^ currents were not different between control renal and non-renal DRG neurons. Phasic neurons exhibited higher Na^+^ and K^+^ currents than tonic resulting in shorter APs (3.7 ± 0.3 vs. 6.1 ± 0.6 ms, *P* < 0.01). In neurons incubated with CXCL1, Na^+^ and K^+^ peak current density increased in phasic (Na^+^: − 969 ± 47 vs. − 758 ± 47 nA/pF, *P* < 0.01; K^+^: 707 ± 22 vs. 558 ± 31 nA/pF, *P* < 0.01), but were unchanged in tonic neurons. Phasic neurons exposed to CXCL1 showed a broader range of Na^+^ currents ([− 365– − 1429 nA] vs. [− 412– − 4273 nA]; *P* < 0.05) similar to tonic neurons. After CXCL1 exposure, significant changes in phasic neurons were observed in sodium activation/inactivation as well as a wider distribution of Na^+^ currents characteristic of tonic neurons. These findings indicate a subgroup of tonic neurons besides mere tonic or phasic neurons exists able to exhibit a phasic activity pattern under pathological conditions.

## Introduction

It is still not completely understood how renal innervation influences systemic autonomous nerve activity [18]. However, numerous publications suggest an important role of renal afferent innervation [1–3, 6, 13, 17].

In the study of the afferent renal innervation in rat models, we use an in vitro model allowing for the investigation of dorsal root ganglion (DRG) neurons that originally had renal afferent nerve projections in vivo [9, 10, 21, 22] since single afferent renal nerve bundles are delicate and difficult to investigate in vivo.

With the help of this model, DRG neurons can be categorized by their firing pattern as “tonic” (high activity) or “phasic” (low activity) [9, 10, 21, 22]: Neurons that show sustained action potential (AP) generation during depolarizing current injection are defined as *tonic neurons*. In contrast, neurons showing only initial and transient AP generation during current injection were defined as *phasic neurons*. In neurons from healthy control animals, tonic neurons were always more abundant in samples of neurons with renal projections than respective phasic neurons.

In models of nephritis [22] and of renovascular hypertension in rats [21] as well after incubation with an inflammatory cytokine [7], the number of phasic neurons increased significantly at the expense of tonic neurons in samples of renal afferent DRG neurons. In nephritis, this finding correlated with a decreased afferent nerve activity accompanied by an increase in central sympathetic outflow to the kidney. Hence, an increase in phasic neurons in vitro (primary cell culture) is likely a reflection of a decreased afferent nerve activity in vivo*,* supporting the idea that afferent renal nerves cannot be unequivocally judged as sympathoexcitatory regulators. It seems that there is rather a loss of sympathoinhibition than mere sympathoexcitation [8, 22].

We investigated not only neurons from DRGs Th11-L2, which had projections to the kidney, but also non-renal neurons that project mainly to the hindlimbs [12]. It turned out in investigations assessing proton sensitivity [9] or the effects of the cytokine CXCL1 [7] on the activity of DRG neurons that the phasic firing pattern exhibited by non-renal neurons was more or less unaffected by any interference like cytokine exposure. The relative abundance of tonic neurons is obviously quite specific for the kidney [19], although the meaning and consequences are not yet clear.

CXCL1 exhibits neutrophil chemoattractant activity [16, 24], eliciting its effects through the chemokine receptor CXCR2 [26] in inflammation [32]. It was of importance in the pathogenesis of models of acute renal failure [14] and in nephrotoxic nephritis of rats [31]. It is also likely to be important in renal inflammation due to other disease processes, such as malignant hypertension [11].

In progressive chronic kidney disease, upregulation of target mRNAs participating in inflammatory pathways included CXCL1 [23]. The CXCR1/CXCR2 antagonist G31P inhibited nephritis in a mouse model of uric acid nephropathy [30]. Experimental glomerulonephritis driven by polymorphonuclear cells was accompanied by an increased renal expression of CXCL1 [27].

CXCL1 and CXCL2 inhibited the axon outgrowth in adult rat dorsal root ganglia neurons, an effect that was mediated by CXCR2 [5]. Furthermore, CXCL1 activated TRPV1 in dorsal root ganglion neurons by mechanisms dependent on CXCR2 receptors and actin filaments [4]. Overexpression of the G protein-coupled kinase 6 attenuated neuropathic pain via suppression of CXCR2 in rat dorsal root ganglion [33]. The chemokine CXCL1 increased sodium currents and neuronal excitability in rat dorsal root ganglia neurons [28]. In the respective study, the overexpression of the CXCR2 receptors was reported on almost all respectively stained neurons [28].

We now wanted to test the hypothesis that the reduction of afferent firing activity seen in inflammation is dependent on altered sodium currents in neurons with tonic firing patterns, thus switching tonic to phasic firing patterns. We used our cell culture model for the investigation of DRG neurons with and without afferent projections from the kidneys. We harvested Th11-L2 DRG neurons that were later incubated with the cytokine CXCL1 in culture [7]. The current clamp mode was used to investigate neuronal firing patterns (i.e., phasic vs. tonic). The voltage clamp was used to investigate membrane currents due to activation and inactivation of voltage-gated sodium channels (Na*v*) and activation of voltage-gated potassium channels (K*v*). As previously, we used the neuronal cell body as a substitute for its projection to the kidney or other organs. Despite certain limitations of this approach, it resulted in findings that could be correlated with renal afferent activity in vivo [22].

## Material and methods

Thirty-five Sprague–Dawley rats (Charles River, Kisslegg, Germany) weighing 180–250 g (9 to 12 weeks of age) were maintained in our animal facility at 24 ± 2 °C. They were fed a standard rat diet (no. C-1000, Altromin, Lage, Germany) containing 0.2% sodium by weight and had free access to tap water. All procedures performed on animals were done in accordance with the guidelines of the American Physiological Society and in compliance with NIH Guide for Animal Care and Use in Laboratory Practice. They were approved by the local government agency (Regierung von Mittelfranken).

### Labeling of renal afferent neurons

To identify DRG cells that project to the kidneys, the dicarbocyanine dye (DiI, 1, 1′ dioleyl-3, 3, 3′ tetramethyl-indocarbocyanine methansulfonate, in EtOH; Molecular Probes®, Darmstadt, Germany) was applied to both kidneys by subcapsular application (5 μl of a solution of 10 g DiI/L) under isoflurane anesthesia (2–3% Forene®, Abbott AG, Baar, Switzerland; N_2_O, O_2_, Linde Gas Therapeutics, Unterschleißheim, Germany) as described previously [10, 20]. The renal hila and lower renal poles were exposed through a small flank incision. Rats were given buprenorphine (0.05 mg/kg, Temgesic®, RB Pharmaceuticals, Berkshire, UK) for post-surgical analgesia.

### Neuronal cell culture

One week after the labeling procedure, the rats were again anesthetized with isoflurane, euthanized by decapitation, and DRGs from Th11-L2 were collected [9, 20]. Neurons were isolated by mechanical and enzymatic dissociation, as described previously [20]. The ganglia were incubated with collagenase IA (2 mg/ml C9891, Sigma Aldrich, Munich, Germany in DMEM, PAA Laboratories GmbH, Linz, Austria) for 1 h in 5% CO_2_ at 37 °C. Enzymatic dissociation was terminated by replacing collagenase-containing DMEM with fresh DMEM + cultural medium (DMEM + , i.e., DMEM plus 10% FCS, 1% penicillin/streptomycin, and 0.1% insulin). Tissue digestion was stopped by FCS. Ganglia were triturated using sterile Pasteur pipettes (Sigmacote®; Sigma-Aldrich, Munich, Germany) to dissociate individual cells. After centrifugation at 100 rcf, cells were resuspended in 10 ml DMEM + and centrifuged once more. The pellet was resuspended in 1.8 ml DMEM^+^, and cells were plated on glass coverslips coated with poly-L-lysine. The coverslips were cultured in DMEM^+^ for one day before electrophysiological experiments.

### Adding chemokine (CXCL1) to cultivated neurons

CXCL1 (1.5 nmol/l, CRG502B, Cell sciences, Canton, MA) was added to every second culture preparation, and cells were allowed to incubate overnight for 20 h, as described previously [7]. Recordings were made within 30 h of plating. In the harvested DRG neurons that were cultured and underwent further investigation, the largest group of cells was represented by medium capacitance and medium size, as previously described [9].

Patch clamp recordings were obtained using a pipette solution containing substances as outlined in Table 1 (*solution 1*: pipette solution). Recordings were conducted in whole-cell mode using pipette resistances of 1–6 MΩ. Patch clamp recordings were obtained with an Axopatch 200B amplifier (Axon Instruments, Foster City, CA). Data were sampled at 20 kHz for voltage and current clamp and analyzed with pClamp® 10.2 (Axon Instruments, Foster City, CA).

**Table 1:** Composition of the solutions used for the patch clamp experiments (see text for more details)

		*Solution 1, intracellular(pipette)*	*Solution 2, extracellular(recordingchamber)*	*Solution 3, extracellular(recording chamber)*
*Substances*	*g/mol*	*Concentration in mmol/l*		
NaCl	58.4	5	140	-
KCL	74.6	140	5	-
MgCl_2_·6H_2_O	203.3	2	1	2
CaCl_2_·2H_2_O	147.0	1	2	1.6
HEPES	238.3	10	10	10
D( +)Glucose	180.1	-	10	15
Natriummethansulfonat	118.1	-	-	30
Tetraethylammonium-chloride	165.7	-	-	100
4-Aminopyridin	94.1	-	-	5
CdCl_2_	-	-	-	0.2
EGTA	380.3	11	-	-
Mg-ATP	507.2	2	-	-
Na-GTP	523.2	0.3	-	-
pH (KOH)		7.35	7.35	7.35

For current clamp protocols, we used extracellular solution 2 (see Table 1). In order to avoid saturation of the patch clamp amplifier, we used the sodium (and potassium)-reduced solution 3 (see Table 1) for the voltage clamp protocols. The liquid junction potential for extracellular solution 2 was approximately 5 mV, and for extracellular solution 3, it was approximately 2.5 mV, as calculated using the open-source software described by Marino et al. [15]. LJP was accounted for during recordings.

**Fig. 1:**
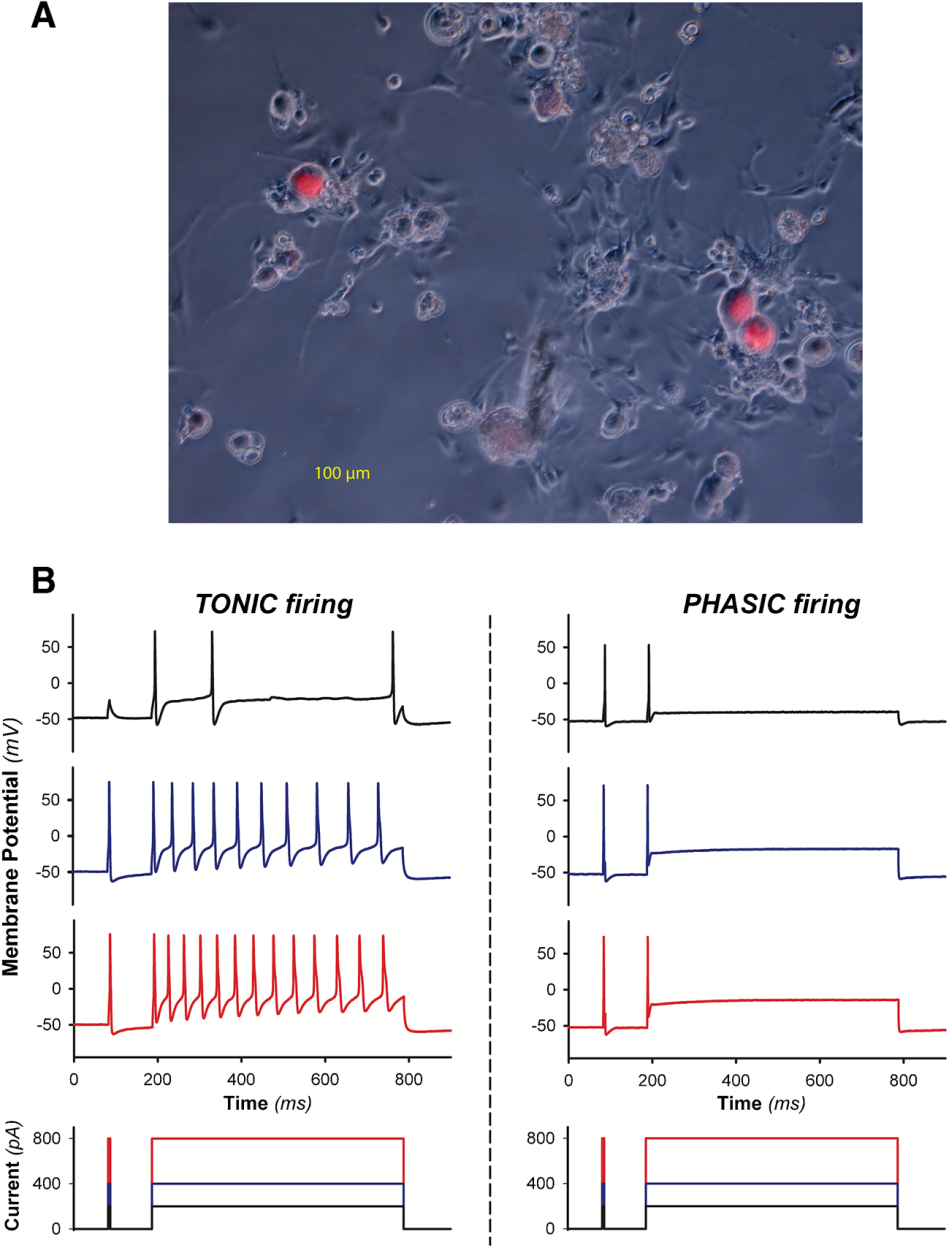
(**a**) Photomicrograph (magnification 150 ×) of labeled DRG neurons on a coverslip under the patch clamp microscope with laser excitation. To achieve the necessary contrast to identify the respective cell types (neurons, fibrocytes, a.o.), phase-contrast microscopy is used as standard. The image is an overlay of a phase-contrast image and the respective DiI fluorescence. (**b**) Representative original traces from current clamp experiments: Tonic DRG neurons (left upper panel) show sustained action potential firing during to supra-threshold current injection, whereas phasic DRG neurons show only initial AP firing. Lower panels display the respective current clamp protocol

Only neurons with a resting membrane potential below − 40 mV were investigated. Cells that stained brightly for DiI to laser excitation (540 nM) were considered renal afferent neurons (see Fig. 1a). Non-renal neurons from the same cultures, i.e., those that showed no DiI staining at all, were also tested [7]. All recordings were done at room temperature, i.e., 22 ± 2 °C.

Cell capacitance was compensated manually, and cell parameters (size, capacitance, and resistances) were documented.

### Current clamp protocols – effects of CXCL1

In current clamp experiments using extracellular *solution 2*, we determined DRG firing patterns utilizing a current clamp approach adopted routinely in our experiments [7, 9, 20–22]. PClamp® 10.2 (Axon Instruments, Foster City, CA) was used to control current pulse generation to record membrane potentials and for offline data analysis. Action potentials were induced by rectangular current pulse injections as follows: A 5 ms pre-pulse, followed by a 600 ms pulse with an inter-pulse delay of 100 ms, was delivered in three consecutive trains of increased intensity (40–400 pA, 400–4.000 pA, 4000–12.000 pA), in 10 consecutive steps (5.16 s each). We categorized DRG neurons as “*tonic*” or “*phasic”* neurons as described previously [7, 9, 20–22]. In short, neurons exhibiting 1 to 4 action potentials (AP) only at the beginning of the supra-threshold current injection were regarded as *phasic* (low activity) neurons, those exhibiting more than four APs as throughout the whole current injection period were regarded as *tonic* (high activity) neurons (see Fig. 1b for illustrative original traces).

### Voltage clamp protocols – effects of CXCL1

In the voltage clamp experiments using extracellular *solution 3*, neurons underwent a voltage clamp protocol to investigate the effects of CXCL1 on neuronal membrane currents. Specifically, we focused on fast sodium and prolonged potassium currents. Pre-defined clamping voltage steps were used for this purpose. Initially, a subthreshold clamp potential of − 80 mV was applied so that no voltage-dependent sodium channels (Na*v*) could be activated. After 50 ms, the clamping voltage was increased stepwise by 10 mV each from − 100 to + 60 mV in successive independent runs (17 voltage steps of 50 ms in 17 successive runs) to measure activation of voltage-gated sodium channels [Na*v*-Act] and potassium channels [K*v*-Act] (see Fig. 3b,c for illustration). This was followed by another 50 ms with a supra-threshold clamping voltage of − 20 mV that theoretically activates sodium channels to give some insight into the inactivation of voltage-gated sodium channels [Na*v*-Inact] (in Fig. 3b,c). This voltage clamp protocol was repeated three times. To avoid saturation of the patch clamp amplifier, the extracellular standard *solution 2* was completely replaced by the sodium (and potassium)-reduced extracellular *solution 3* (see Table 1).

The inactivation current curves were obtained by measuring the inward current at − 20 mV clamping voltage: If the immediately preceding clamping voltage level was maximally subthreshold (e.g., − 100 mV), a maximum inward current could be expected due to activation of voltage-gated sodium channels (Na*v*) in this condition. If, on the other hand, the preceding clamping voltage level was supra-threshold, more and more sodium channels would be inactivated as the previous clamping voltage increased so that the resulting inward current in this phase of the clamping voltage protocol would get lower and lower until finally no inward current would be measurable once the maximum inactivation rate has been reached. Thus, the reduction of inward current was correlated to Na*v* inactivation.

### Data analysis

Data were analyzed using standardized procedures (pClamp 10.2 and Clampfit 10.2 software package). In accordance with a published method [25], the threshold was defined as the point where the membrane potential started to increase. The corresponding membrane voltage at that point was defined as the threshold voltage. We used the method of maximal slope assuming that the action potential threshold is the voltage where the first temporal derivative V’ shows a maximal rate of change with respect to initial curve V, i.e., the maximal slope of V’ versus V according to *f* = dV/dt = V’ using the analyzing functions of Clampfit 10.2. The action potential duration was measured at a threshold voltage level in milliseconds. These parameters were analyzed in the first action potential induced by the second-lowest supra-threshold current injection. The analysis of maximum action potential frequency was done at the current step, which induced the highest frequency without distortion of the action potentials.

All data underwent normality testing (Shapiro–Wilk) to assess the distribution of data. *T*-tests were applied for the comparison of two groups or treatments, respectively, with normal distribution at defined time points or at defined clamp voltage levels in the protocols; otherwise, non-parametric testing (Mann–Whitney rank sum test; MWrst) was used. To test repeated membrane current responses due to the 17 runs of the voltage clamp protocol, we used one-way ANOVA with multiple comparisons vs. control or baseline, i.e., the respective membrane current at − 80 mV clamp voltage. We used Dunnett’s test in this context. For data sets that failed the normality testing (Shapiro–Wilk), the Kruskal Wallis RM ANOVA on ranks was used.

The *z*-test was used to test for significant differences in the distribution of characteristics of the DRG neurons (e.g., renal, non-renal, phasic, tonic, incubation with or without CXCL1). Statistical significance was defined as *P* < 0.05 (two-sided). Data are presented graphically as group means ± SEM or median [min/max]. SigmaPlot 14 and SigmaStat 3.5 (Systat Software, Erkrath, Germany) were selected for statistical analysis and graphical display.

## Results

308 DRG neurons were included in the analysis. Slightly more than half of the evaluated neuron cohort (56.5% [174/308]) glowed clearly in an orange hue under the laser microscope (DiI-positive) and were accordingly classified as “renal afferent” (see Fig. 1a for illustration). In contrast, the non-orange glowing DiI-negative cells (43.5% [134/308]) were classified as “non-renal afferent” neurons, as described above. 184 CXCL1-incubated DRG neurons were included in the analysis, of which 54.9% (101/184) were DiI-positive, thus renal afferent neurons. In contrast, 45.1% (83/184) of CXCL1-incubated neurons were DiI-negative and thus classified as non-renal afferent neurons.

**Fig. 2:**
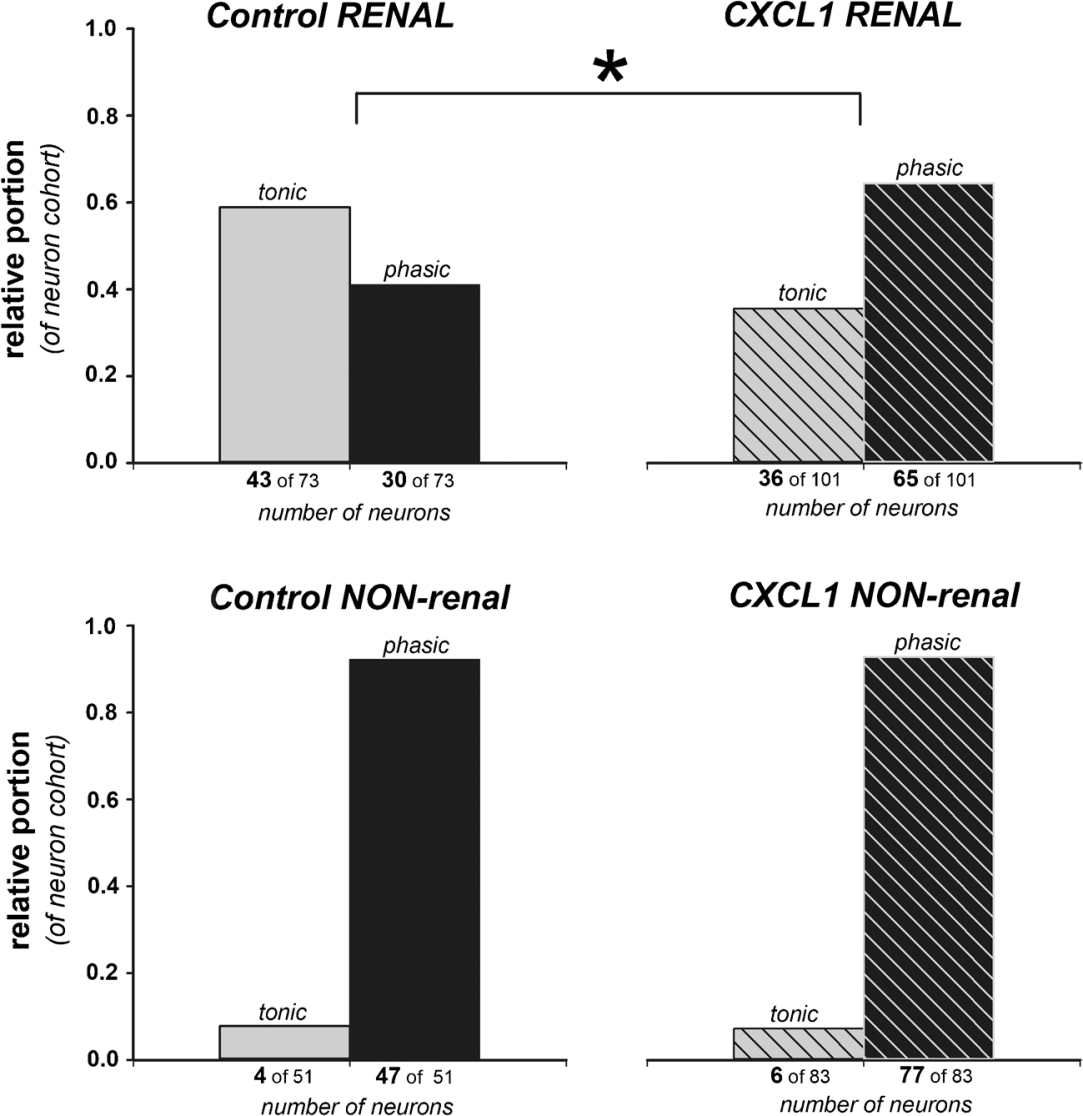
Relative portion of neuron properties (*tonic vs. phasic* firing pattern) in different DRG neuron cohorts (*renal vs. non-renal*): 58.9% of renal afferent DRG neurons showed tonic firing pattern under control conditions due to supra-threshold current injection. CXCL1 incubation decreased tonic firing to 35.6% in favor of neurons that show of phasic firing pattern; *P < 0.05 (*z*-test). In non-renal afferent DRG neurons, no such effect was seen

When *phasic* and *tonic* control neurons (see also Fig. 1b for illustration) were compared, a small but statistically significant difference in membrane potential was found (phasic, *n* = 77: 52.0 mV [− 55.0– − 0.0] *vs.* tonic, *n* = 48: − 49,0 mV [− 52.5– − 46.0]; *P* < 0.001). Due to the fact that tonic firing was nearly exclusively found in the cohort of renal DRG neurons (see Fig. 2), we also compared renal and non-renal DRG neurons, irrespective of the property “tonic or phasic.” However, there was no difference when *renal* and *non-renal* control neurons were compared (renal, *n* = 73: − 51,0 [− 64,0– − 39.0] *vs.* non-renal, *n* = 51: 52,0 [− 63,0– − 39,0]; *P* = 0.718). Furthermore, incubation with CXCL1 or vehicle did not alter the resting membrane potential of the respective neuron cohorts (*phasic* vs. *tonic*) that were finally analyzed.

### Tonic and phasic response pattern – AP generation in controls and after CXCL1 incubation (Fig. 2 and Table 2)

**Table 2:** Electrophysiological characteristics of dorsal root neurons with afferents axons from the kidneys

		Tonic neurons		Phasic neurons	
		*Renal*	*Non-renal*	*Renal*	*Non-renal*
Control	AP duration *(ms)*	5.2 [2/28] 6.2 ± 4.2 (0.62) *n* = 44 skew: 3.328 *#1: P* < *0.05* *MWrst*	3.7 [2/9] 4.6 ± 3.4 (1.7) *n* = 4 skew: 1.143 *P* = *ns* *t-test*	4.3 [1.7/18] 5.4 ± 4.0 (0.7) *n* = 30 skew: 1.806 *#3: P* < *0.001* *MWrst*	2.4 [1.5/7] 2.7 ± 1.0 (0.15) *n* = 46 skew: 2.438 *#6: P* < *0.001* *MWrst*
	AP threshold *(mV)*	− 23.8 [− 41/ − 5.4] − 23.7 ± 8.8 (1.96) *n* = 20 skew: 0.154 *#2: P* = *0.01* *t-test*	− 41.2 [− 67/ − 24] − 43.5 ± 18.1 (9.1) *n* = 4 skew: − 0.690 *P* = *ns* *t-test*	− 45.5 [− 70/ − 11] − 45.3 ± 12.5 (2.3) *n* = 30 skew: 0.665 *#4: P* < *0.001* *MWrst*	− 39.0[− 48/ − 30] − 38.7 ± 3.9 (0.83) *n* = 22 skew: 0.094 *#7: P* < *0.001* *MWrst*
	AP frequency *(AP/600 ms)*	17.0 [6/33] 16.6 ± 8.0 (1.2) *n* = 44 skew: 0.108 *P* = *ns* *MWrst*	15 [9/23] 15.5 ± 7.0 (3.5) *n* = 4 skew: 0.137 *P* = *ns* *t-test*	1 [1/4] 1.7 ± 0.9( 0.2) *n* = 30 skew: 1.420 *#5: P* < *0.001* *MWrst*	1 [1/2] 1.2 ± 0.4 (0.05) *n* = 48 skew: 1.847 *#8: P* < *0.01* *MWrst*
CXCL1	AP duration *(ms)*	4.6 [2/9] 4.6 ± 1.7(0.3) *n* = 36 skew: 1.006 *#1: P* < *0.05* *MWrst*	* 3.7 [1.6/7] 4.0 ± 2.2 (0.8) *n* = 7 skew: 0.336 *P* = *ns* *t-test*	2.0 [1/9.6] 2.8 ± 1.8 (0.2) *n* = 65 skew: 1.913 *#3: P* < *0.001* *MWrst*	2.0 [1.2–4.6] 2.2 ± 0.7 (0.08) *n* = 76 skew: 1.794 *#6: P* < *0.001* *MWrst*
	AP threshold *(mV)*	− 16.4 [− 46/ − 3] − 16.8 ± 9.0(1.5) *n* = 36 skew: − 1.012 *#2: P* < *0.01* *t-test*	− 31.9 [− 37/ − 7.6] − 23.9 ± 14.2 (5.4) *n* = 7 skew: 0.355 *P* = *ns* *t-test*	− 34.0 [− 55/ − 3] − 31.2 ± 10.6 (1.3) *n* = 22 skew: 1.017 *#4: P* < *0.001* *MWrst*	34.7 [− 43/ − 14] 33.4 ± 6.82 (0.8) *n* = 76 skew: 0.988 *#7: P* < *0.001* *MWrst*
	AP frequency *(AP/600 ms)*	17.0 [5/30] 15.7 ± 7.2(1.2) *n* = 36 skew: − 0.057 *P* = *ns* *MWrst*	14 [6/22] 14.4 ± 6.3 (2.4) *n* = 7 skew: − 0.458 *P* = *ns* *t-test*	1 [1/3] 1.1 ± 0.4 (0.05) *n* = 65 skew: 3.928 *#5: P* < *0.001* *MWrst*	1 [1/2] 1.0 ± 0.2 (0.02) *n* = 71 skew: 5.827 *#8: P* < *0.01* *MWrst*

Only effects of CXCL1 incubation within respective neuron cohorts are considered here. Each cell of the table shows respective data as median [min/max], mean ± standard deviation (SEM); *n* gives the number of neurons that were included in the analysis; Skewness (skew) is given to describe the data distribution. # indicates significant differences; the numbers that follow “#” mark the respective individual comparison, i.e., each number (1–8) following the “#” appear twice in the table and denotes the data pair that has been compared. Furthermore, the statistic test used for comparison is shown as dependent on the result of the Shapiro–Wilk test, i.e., either the *t*-test for normally distributed data or the Mann–Whitney rank sum test (MWrst) for not normally distributed data

In the control cohort of DRG neurons *with renal projections* (*n* = 73), current injection revealed a higher portion of neurons with tonic response patterns (43 of 73 = 58,9%) compared to phasic ones (30 of 73 = 41,1%). However, in the neuron cohort *with renal projections* that were incubated with CXCL1 (*n* = 101), the *tonic-to-phasic* response ratio significantly changed in favor of phasic neurons (65 of 101 = 64,4%), with less tonic neurons (36 of 101 = 35,6%) showing a tonic response pattern. No such changes were observed in the non-renal DRG neuron cohort due to incubation with CXCL1, where the phasic response pattern predominated (see Fig. 2).

CXCL1 incubation reduced AP duration and increased the AP threshold to more positive values in tonic renal and phasic neurons. However, pretreatment with CXCL1 decreased AP frequency only in phasic neurons. No statistically significant effects of CXCL1 incubation were seen in the small groups of non-renal tonic neurons (see Table 2).

### Tonic vs. phasic DRG neurons – membrane currents (Fig. 3)

**Fig. 3:**
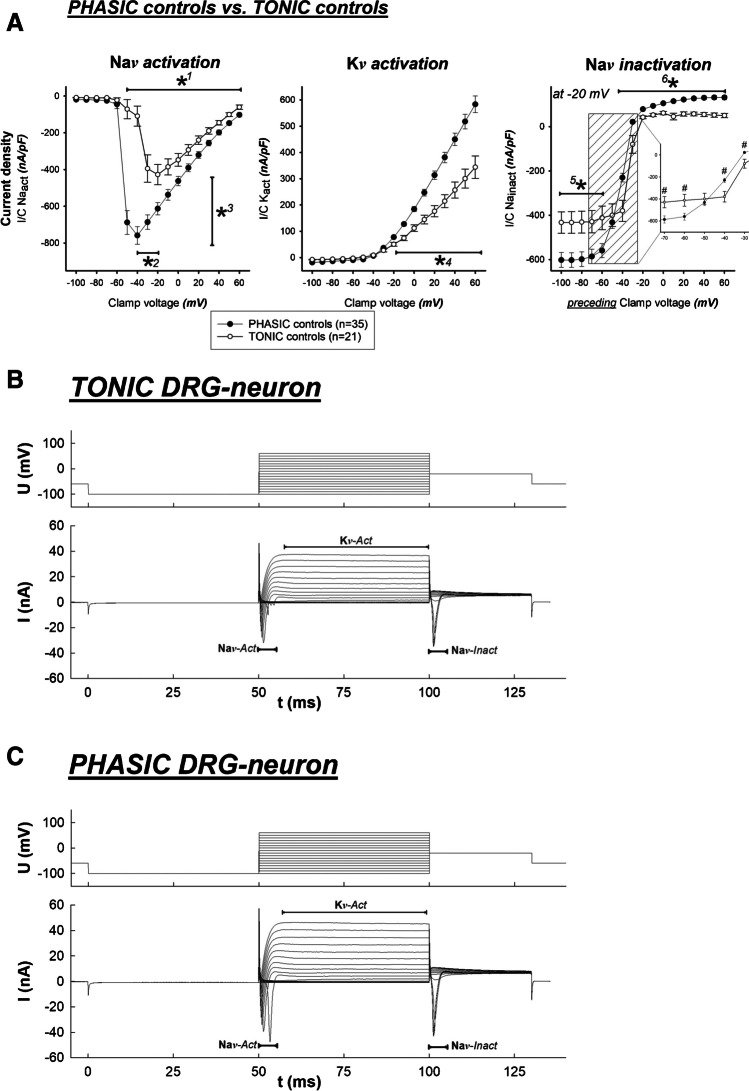
(**a**) Phasic control neurons (•) showed greater depolarising Na^+^ peak inward currents (*^1^, *^3^ *P* < 0.001) at lower clamp voltage (*^2^ *P* < 0.001), i.e., stronger Na*v* activation, higher repolarising K^+^ outward currents (*^4^ *P* < 0.001), i.e., stronger K*v* activation, and stronger Na*v* inactivation (*^5^, *^6^ *P* < 0.001) as compared to tonic neurons (〇). The inlay graph in the right-sided panel displays the relevant interval of the Na*v* inactivation. Note the intersecting curve progressing due to the enhanced Na*v* inactivation of phasic neurons as compared to tonic neurons (^#^*P* < 0.001). (**b**) The upper panel displays an overlay of the voltage clamp protocol (clamp voltage [mV] versus time [ms]), 17 sweeps with incrementally depolarizing clamp voltages (interval between 50 and 100 ms), the lower panel displays the overlay of resulting membrane currents of a representative *tonic* DRG neuron. Amplitudes of sodium activation current (Na*v*-Act) and potassium activation current (K*v* Act) are smaller than in tonic DRG neurons (see Fig. 3c for comparison). (**c**) The voltage clamp protocol is identical to that in Fig. 3b; the resulting membrane currents of a representative *phasic* DRG neuron look rather similar, however, with larger amplitudes of Na*v*-Act and K*v*-Act. Sodium inactivation (Na*v*-Inact) cannot be assessed from overlayed tracings

Figure 3 shows the “tonic vs. phasic” comparison of membrane current densities with no distinction made between renal or non-renal (DiI-pos or DiI-neg). This and the following graphical representations of the membrane current densities follow a uniform structure, in which current density is the measured membrane current related to the neuronal cell capacity, which is dependent on the cell membrane surface and is thus related to cell size: From left to right, the sodium activation current densities (Na*v* activation), the potassium activation current densities (K*v* activation), and the sodium inactivation current densities (Na*v* inactivation) are shown (nA/pF) according to the order of occurrence of the respective currents due to the clamp voltage steps in the voltage clamp protocol outlined above. In the context of Na*v* inactivation graphs, it has to note that the plotted current density values were uniformly obtained at a clamp voltage of − 20 mV; however, the *x*-axis labeling in the Na*v* inactivation plots denotes the immediately preceding clamp voltage before the switch to − 20 mV. Only those data sets were included in the analysis, where all three consecutive current components were completely free of artifacts.

Significant differences between tonic and phasic neurons were found in all measured current components (Na*v* activation, K*v* activation, Na*v* inactivation): Phasic neurons showed a greater (inward) peak Na^+^ current density in Na*v* activation. This peak was measured at a lower clamp voltage (− 47.4 ± 1.9 mV) compared to the lesser Na^+^ peak current density of tonic DRG neurons, which was measured at a significantly less negative clamp voltage (− 28.3 ± 2.8 mV). The K*v* activation current density was also significantly higher in the phasic cells at clamping voltages above − 20 mV. The Na*v* inactivation curves were also significantly different. Phasic cells exhibited, in congruence with Na*v* activation, a significantly stronger inward current when coming from a clearly subthreshold clamp voltage, but from a (preceding) clamping voltage of − 50 mV and less, a significantly more pronounced, i.e., “faster,” Na*v* inactivation was found, as compared to the tonic cells. Maximum Na*v* inactivation was reached from about − 30 mV for the phasic ones and from about − 20 mV for the tonic ones. These findings correlate with the findings from the current clamp protocols: Faster Na*v* activation and inactivation should result in shorter action potential durations. The slightly positive ionic current between − 20 and 60 mV is likely to be carried by potassium outflow. These findings also correlate with those of K*v* activation: higher values for phasic neurons, slightly lower values for tonic neurons, so that phasic neurons are also likely to repolarize faster compared with tonic neurons.

### Phasic and tonic DRG neurons – renal versus non-renal (Fig. 4)

**Fig. 4:**
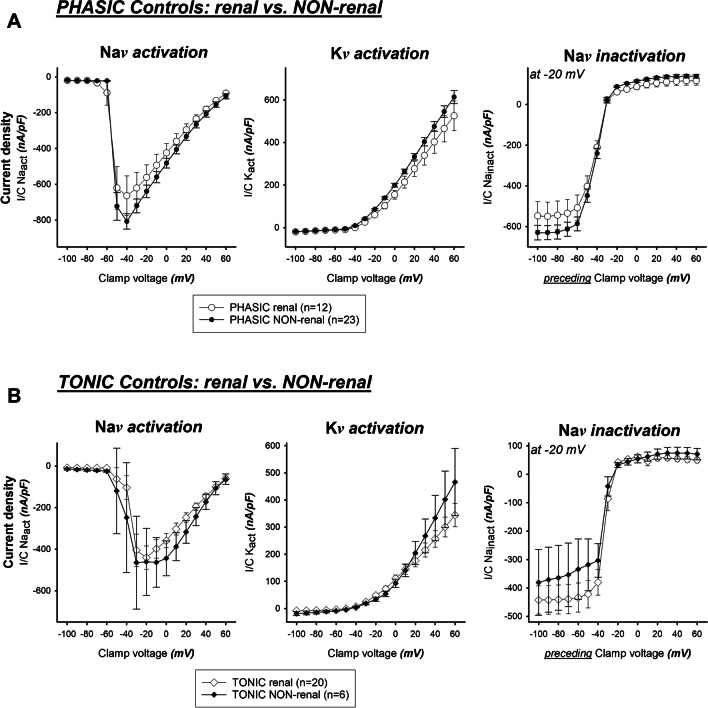
(**a**) Phasic renal (〇) and non-renal (•) neurons did not show significant differences when respective membrane currents of these cohorts were compared. (**b**) Tonic renal (◇) and non-renal (◆) neurons did not show significant differences when respective membrane currents of these cohorts were compared

In a further analysis step, separate comparisons of “phasic renal vs. phasic non-renal” and “tonic renal vs. tonic non-renal” neurons were made in the group of non-incubated control neurons. No significant differences with respect to the property “renal vs. non-renal” were found.

### Phasic DRG neurons – CXCL1 versus controls (Fig. 5)

**Fig. 5:**
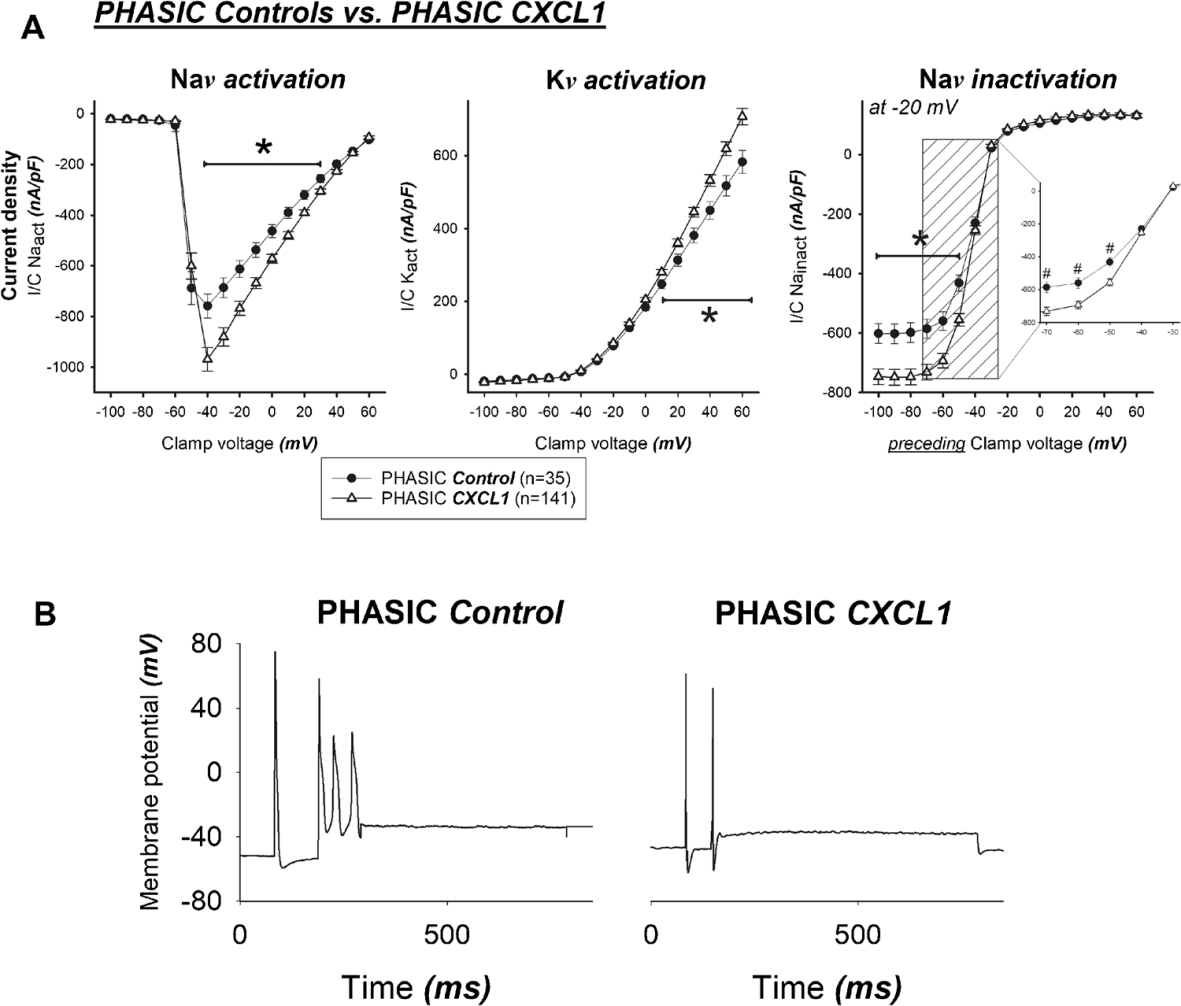
(**a**) After incubation with CXCL1, phasic neurons (△) showed greater depolarising Na^+^ peak inward currents (i.e., Na*v* activation), higher repolarising K^+^ outward currents (i.e., K*v* activation), but weaker Na*v* inactivation, as compared to phasic controls (•) *P < 0.001. The inlay graph in the right-sided panel displays the relevant interval of the Na*v* inactivation. Note the approaching curve progressing due to the attenuated Na*v* inactivation of phasic neurons as compared to tonic neurons. ^#^*P* < 0.001. (**b**) Displayed are representative membrane voltage traces due to a supra-threshold current injection of a phasic control neuron (left panel) and phasic neuron after CXCL incubation (right panel). The low phasic activity seems to become even more silenced after CXCL1 incubation

Since there were no significant differences in the membrane currents of the control neurons with respect to the comparison “renal vs. non-renal,” the total cohorts of phasic and tonic neurons were analyzed separately in the context of CXCL1 incubation.

In the phasic neuron group, incubation with CXCL1 resulted in a significantly stronger Na*v* activation current density at clamp voltages between − 40 and + 40 mV, an increase in K*v* activation currents at clamp voltages from − 10 to + 60 mV, and an increase in Na*v* inactivation currents at clamp voltages from − 100 to − 60 mV equivalent to sodium activation. At the same time, however, decreased Na*v* inactivation is evident at (pre)-clamp voltages between − 60 and − 50 mV.

### Tonic DRG neurons – CXCL1 versus controls (Fig. 6)

**Fig. 6:**
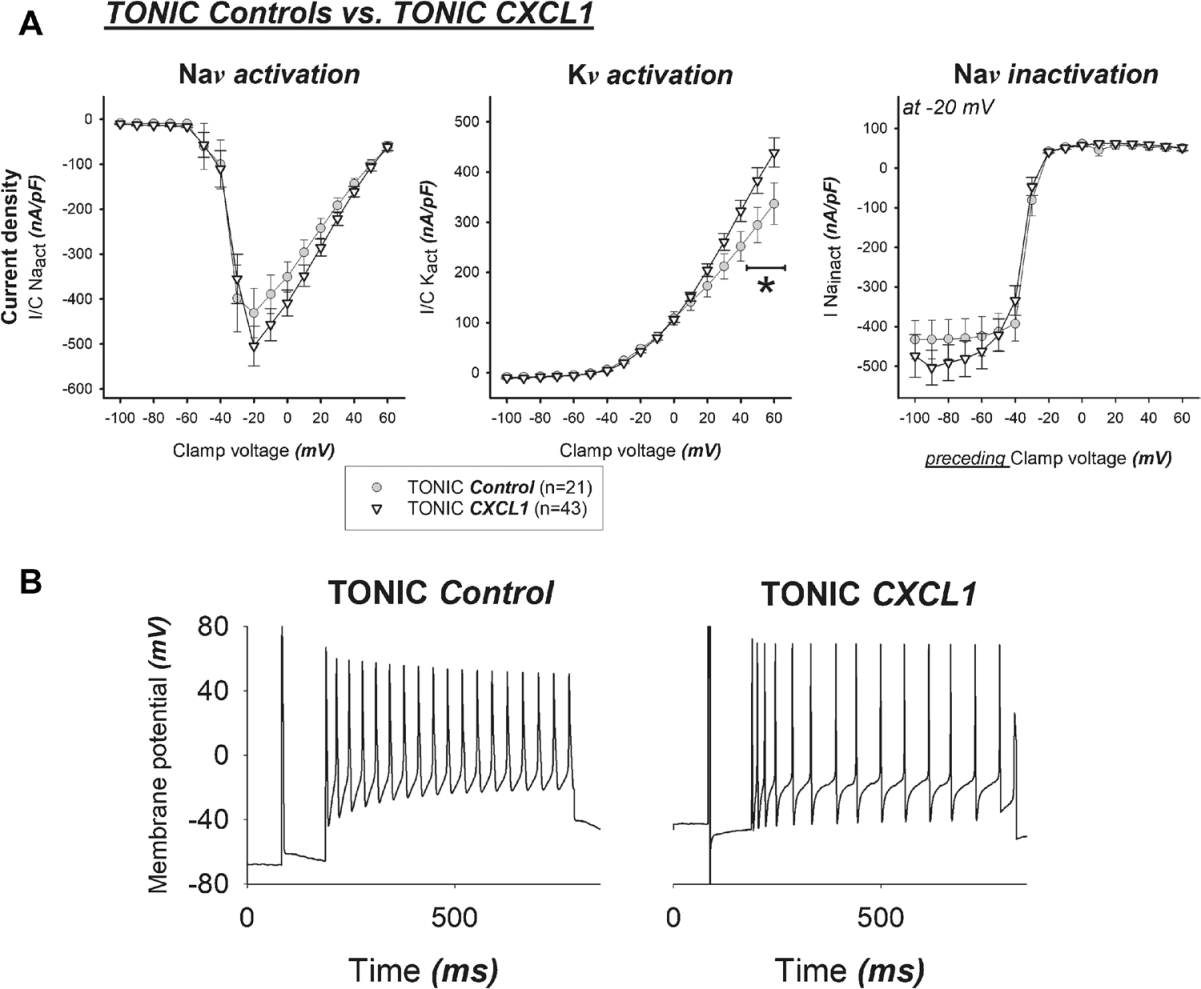
(**a**) After incubation with CXCL1, the “remaining “ tonic neurons (▽) did not show any significant differences in Na*v* activation Na*v* inactivation when compared to tonic controls (•). However, K*v* activation was increased. *P < 0.001. (**b**) Displayed are representative membrane voltage traces due to a supra-threshold current injection of a tonic control neuron (left panel) and tonic neuron after CXCL incubation (right panel). Action potential duration and frequency is reduced after CXCl1 incubation

In contrast to phasic neurons, incubation with CXCL1 in the tonic neuron group resulted in remarkably fewer effects. No significant effect of CXCL1 on Na*v* activation or inactivation was found. Slow potassium outward currents were not significantly affected by CXCL1 incubation when the absolute voltage data measured were analyzed without reference to cell capacitance. In terms of current density, however, an increase in outward potassium flow was also seen in tonic cells. Thus, the reduced cohort of the remaining tonic neurons appeared to be relatively resistant to the influence of CXCL1, at least with respect to sodium current density.

### Na^+^ current distribution: CXCL1 effects – tonic versus phasic DRG neurons (Fig. 7)

**Fig. 7:**
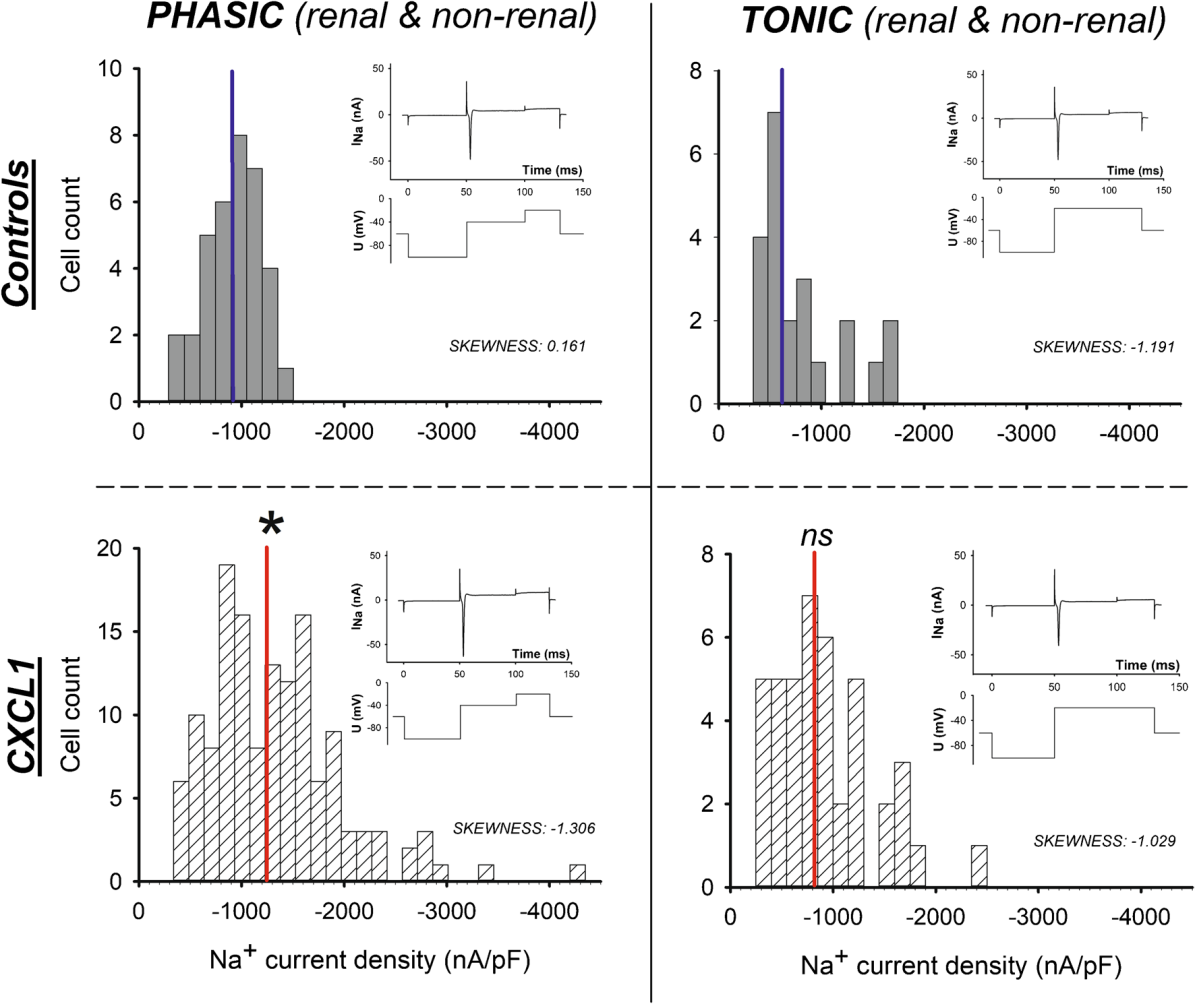
Na^+^ inward currents in phasic control DRG neurons showed a normal distribution (Shapiro–Wilk test passed), while Na^+^ currents in tonic DRG neurons are not normally distributed even under control conditions (SP-test failed). After CXCL1 incubation, phasic DRG neurons showed a broader range of Na^+^ currents which was no longer normally distributed. The median of Na^+^ inward currents in phasic DRG neurons was significantly higher after CXCL1 incubation, *P < 0.001. This was not the case in tonic DRG neurons

After the analysis of the CXCL1 effect on the membrane currents under the perspective “tonic vs. phasic” and in synopsis with the shifts of the AP firing behavior in the subgroups “renal vs. non-renal,” it turned out that under the influence of CXCL1, on the one hand, the tonic firing behavior was significantly less observed in the renal neuron cohort, but in the remaining tonic neurons, hardly any changes in the membrane currents could be detected. In contrast, the membrane currents in the phasic neurons under CXCL1 showed significant changes in all three phases of the voltage clamp protocol: An increased Na*v* and K*v* activation but a decreased Na*v* inactivation.

Therefore, in Fig. 7, the peak inward sodium currents of the neuron cohorts are plotted as histograms to show the distribution of the absolute measured currents.

The peak inward sodium currents of the phasic, untreated control DRG neurons originated from a normally distributed cohort (Shapiro–Wilk test). Skewness was calculated with 0.161, indicating a normal distribution of the data. However, this was different for tonic cells: Here, the data sets failed normal distribution testing. Skewness was calculated with − 1.191, indicating non-normal distribution. Furthermore, CXCL1 incubation of phasic cells shifted the distribution width to a non-normally distributed pattern similar to that of the non-normally distributed tonic neurons (skewness − 1306). The distribution pattern and width of tonic neurons did not change with CXCL1 incubation (skewness − 1.029).

## Discussion

In this paper, we describe for the first time that the afferent renal innervation comprises at least three different neuron subpopulations: a high-activity *tonic* one, a low-activity *phasic* one, and a special group of *tonic* neurons that can switch to phasic after exposure to the cytokine CXCL1. This conclusion is based on the following findings: The results from the voltage clamp experiments revealed significant changes in the voltage-gated sodium channel (Na*v*) activation and inactivation and in potassium channel (K*v*) activation in phasic neurons as well as a wider distribution of Na*v* activation currents similar to the sodium current distribution seen in tonic neurons. These findings indicate that a subgroup of tonic neurons exists, which switches to a phasic firing pattern under pathological conditions.

We could not fully confirm our hypothesis that the switch from tonic to the phasic pattern in the third subgroup of afferent renal neurons is solely dependent on sodium currents. Sodium currents play an important role, but other ion channels must be involved in addition. Potassium channels will likely play an important role.

Exposure to the cytokine CXCL1 was confirmed to reduce the number of tonic renal neurons in favor of phasic neurons. We observed this phenomenon in several pathological settings, such as nephritis [22], renovascular hypertension [21], and cytokine incubation [7].

In more detail, a comparison of tonic to phasic neurons under control conditions revealed significant differences in these cell populations concerning the voltage clamp protocol if we discuss the electrophysiological parameters obtained: namely, sodium channel (Na*v*) activation (= fast depolarizing inward current), Na*v* inactivation, and potassium channel (K*v*) activation (= repolarizing or hyperpolarizing outward current). Phasic DRG neurons showed greater peak Na^+^ inward currents at a lower threshold, greater peak K^+^ outward currents, and “faster” Na*v* inactivation if these parameters were compared with the tonic neuron population. Of note, these parameters relate to membrane current properties, all of which may help to explain the shorter action potentials of phasic neurons.

In the phasic neuron population, CXCL1 induced larger peak sodium inward currents and larger maximum potassium outward currents, albeit with slightly impaired Na*v* inactivation. In contrast, the “remaining” tonic neurons showed no changes in sodium channel (Na*v*) activation or inactivation, but increased potassium channel (K*v*) activation when current density was analyzed.

However, the effects of CXCL1 on Na*v* inactivation are less imposing than the effect on Na^+^ inward and K^+^ outward currents. As displayed in Fig. 3, phasic neurons show a more effective (i.e., faster) Na*v* inactivation kinetic as compared to tonic neurons, even though they “start” from a larger Na + inward current level. The clear-cut differences in the Na*v* inactivation kinetics are illustrated by the crossing of the respective curves at approximately − 48 mV preceding clamp voltage. In the context of the CXCL1 influence on the Na*v* inactivation of the phasic neurons (see Fig. 5), the effect is less prominent: The inactivation curves of the two groups run reasonably parallel between − 70 and − 50 mV preceding clamp voltage with “phasic CXCL1” well below “phasic controls” (i.e., less pronounced Na*v* inactivation), but approach each other between − 50 and − 40 mV to finally overlap from − 40 to 60 mV. Thus, phasic CXCL1 neurons seem to have a less dynamic Na*v* inactivation kinetic. However, when the maximum “starting” Na + inward current level was taken into account and the relative Na + inactivation was calculated, no clear-cut difference between the groups could be found.

At least, the observed changes in Na*v* inactivation cannot necessarily explain the shortening of action potentials under CXCL1 influence. With slightly reduced or “delayed” Na*v* inactivation, one might expect a prolongation rather than a shortening of action potentials. Thus, the shortening of action potentials in this context is likely to be explained by the increased potassium-driven repolarizing current.

Finally, the distribution of the peak sodium inward currents showed that the phasic neurons under control conditions showed a narrow, normal distribution, which widened markedly under the CXCL1 influence: The sample of phasic neurons lost its normal distribution, shifted to the distribution pattern of the tonic cells, while the latter did not change between control conditions and CXCL1 incubation. The “remaining” tonic neurons, on the other hand, were resistant to these changes in peak Na^+^ inward currents. Nevertheless, the action potentials shortened in the tonic cells as well, which might well be explained by the enhancement of the repolarizing potassium outward currents also detectable in this neuron population.

Published reports indicate that a tonic action potential pattern is associated with specific subtypes of voltage-gated sodium channels (Na*v*) [2, 5]. For example, the TTX-resistant sodium channel Na*v*1.8 exhibits specific kinetics that led to a broader action potential, which could facilitate the development of a continuous, i.e., tonic firing pattern [3–6]. On the other hand, the pattern of phasic action potentials could be explained by the kinetics of TTX-sensitive channels, which mainly cause the AP upstroke and are quickly inactivated, resulting in a narrow action potential [1, 4]. As such, this could be a plausible explanation of our described findings of a change in the activity of different subtypes of voltage-gated sodium channels. This change might either be seen toward increased TTX-sensitive channels or toward less TTX-resistant channels, such as Na*v*1.8 or Na*v*1.9. In a paper by Wang [8], it was shown that incubation of DRG neurons of segments L4/L5 with CXCL1 significantly increased the expression of TTX-sensitive (especially Na*v*1.1 and Na*v*1.7) and, to a lesser extent, TTX-resistant sodium channels (Na*v*1.8), which increased sodium currents. This probably increased the excitability of these cells to CXCL1. In addition, the cells showed an increased propensity for repetitive firing behavior.

In our study, the overall results were somewhat different: The fast sodium influx was likewise increased in phasic cells under CXCL1 influence, which could be indicative of increased excitability. However, we also observed decreased Na*v* inactivation, which should rather promote a prolongation of action potentials, possibly favoring repetitive firing (as in tonic neurons). However, we saw a net effect of a shortening of action potentials and a reduction in tonic, i.e., repetitive firing. In this context, the enhanced potassium currents may play an important role, which may enhance repolarization and, in turn, may also contribute to the shortening of action potentials.

Indeed, there is good evidence in the literature for the influence of CXCL1 also on potassium currents. Using DRG neurons from segments L4/5, it was shown that both the transient and the sustained components of potassium currents were enhanced when the cell culture was incubated with CXCL1 [29]. Here, the transient component attributed a role in the ability of repetitive firing. However, this did not affect all neurons equally, but only the so-called IB4-negative neurons, not the IB4-positive ones.

IB4 positivity describes the ability of neurons to bind the plant lectin isolectin B4 from the African swastika bean (Griffonia simplicifolia). This is used to differentiate the small C-fiber neurons of the dorsal root ganglia into two groups (IB4-pos; IB4-neg). The IB4-negative neurons are the so-called peptidergic neurons, which can produce neurokinins such as substance P and CGRP. They express TRPV1 receptors and ASIC and the trk A receptor, which binds NGF (nerve growth factor). In contrast, IB4-positive neurons are non-peptidergic C-fiber neurons that also express TRPV1 receptors and the P2X3 receptor for ATP, which IB4-negative neurons do not [7]. We have shown in our previous projects that SP and CGRP are increasingly secreted during renal inflammation within the kidney [20, 22]. At the same time, the number of tonic neurons decreased in favor of phasic neurons while the afferent renal nerve activity decreased [22]. The above work on the role of IB4 positivity suggests that the complexity of renal afferent innervation may even be far more complicated than so far discussed: Different afferent renal nerve fibers might be somewhat more specifically involved in paracrine mechanisms, whereas others are mainly transmitting nerve traffic input to the central nervous system.

These considerations must be seen against the background that up to now no good idea exists why such a high physiological expenditure developed with respect to the afferent renal innervation in the healthy organism involving different subgroups of renal nerve fibers.

Eventually, much more research on the basic physiology of renal afferent nerve fibers will be needed before it might be possible to adequately appreciate the role of afferent renal innervation under pathological conditions.

## Data Availability

All data are stored on computers in the laboratory and on additional hard disk media for backup.
